# Ovarian Cysts

**Published:** 1857-11

**Authors:** 


ARTICLE VI.—Translated for the Journal by DR. BYFORD.

*Ovarian Cysts.*

At a recent lively and interesting discussion in the Academie de Medicine of Paris, on the subject of ovarian cysts and their treatment, the following principles were settled and adopted:

1st. Ovarian cysts belong to the list of dangerous diseases, and are usually fatal in their termination. (Cazeau and Huguier.)

2d. The duration of the disease varies from four to six, (Cazeau), ten, or twelve years. (Velpeau.)

3d. It is an error to suppose that the sufferer in the greatest number of cases attains anything like old age, and the younger the patient the more rapidly it terminates.

4th. There are cases that are absolutely incurable, and unmitigable in their nature, viz. the areolar, vesicular, and multilocular non-communicative cysts. (Cruveilheir.)

5th. In many cases can ovarian cysts be cured by proper treatment, and others spontaneously resolve themselves. (Velpeau.)

6th. Spontaneous cures follow as a consequence of rupture of the cyst, but for the most part rupture and effusion are followed by death. (Velpeau.)

7th. Palliative punctures are not dangerous, and sometimes lead to radical cures. They nearly always give great relief, by taking off inconvenient and disagreeable distention. (Velpeau.)

8th. Puncture, followed by injections of the solution of iodine, in the present state of our knowledge, is the most successful procedure with which we are acquainted, in this hitherto incurable affection. (Cazeau.)

9th. It is not advisable to leave a tube or sound in a puncture, as it leads to suppuration almost always.

10th. It is the teaching of experience, that after puncture and the injection of iodine, the puncture should be closed.

11th. Unilocular cysts, without organic alterations of their envelopes, containing serous sero-sanguineous or albuminous fluid, and cysts that have formed as a consequence of extra uterine foetation, admit very frequently of a cure. (Huguier.)

12th. As the discussion reveals the fact, that the puncture with injection is as safe as simple puncture, it is desirable to resort to this measure early as possible, that the patients may have the greatest number of chances in her favor. (Velpeau.)

13th. The best time, therefore, for operating is before the tumor has attained a very large size, or produced any disturbance in the functions of surrounding organs.

14th. Phlegmonous or purulent inflammation of the cyst after puncture, afford grounds for the most gloomy prognosis.

15th. Extirpation of the cyst is the most dangerous of operations, and should not, from any consideration, be performed, particularly as this fact is so well established. (Velpeau.) (Revue Therapeutique du Midi.)

